# A Competency-Based Dental Education Framework for Postgraduate Orthodontic Training: A Comprehensive Review for Effective Curriculum Utilization

**DOI:** 10.7759/cureus.110366

**Published:** 2026-06-06

**Authors:** Hitesh R Sawant, Harsh A Mishra, Parag Gangurde, Sheetal M Jadhav, Priyanka Purohit, Archana Jayakumar

**Affiliations:** 1 Department of Orthodontics and Dentofacial Orthopedics, Bharati Vidyapeeth (Deemed to be University) Dental College and Hospital, Navi Mumbai, IND; 2 Department of Prosthodontics, Bharati Vidyapeeth (Deemed to be University) Dental College and Hospital, Navi Mumbai, IND; 3 Department of Orthodontics, Bharati Vidyapeeth (Deemed to be University) Dental College and Hospital, Navi Mumbai, IND

**Keywords:** competency-based dental education, course outcomes, curriculum mapping, entrustable professional activities, orthodontics, postgraduate orthodontic training, program outcomes

## Abstract

Due to the shift in focus on outcomes, instead of time-bound training, competency-based models have become widely accepted in health professions education. In the context of orthodontic diagnostic procedures and biomechanical challenges, specialty-specific skills such as designing and treatment planning are critical for health practitioners. The latest literature has recognized the core competencies pertinent to postgraduate orthodontic training in the field, yet there continues to be notable variability in program quality, clinical knowledge, and competency tests between training programs. The objective of this review is to design an integrated competency-based dental education (CBDE) model for postgraduate orthodontic training that encompasses the management of students’ competencies by correlating competency domains with specific program outcomes (POs) and course outcomes (COs), introducing entrustable professional activities (EPAs) to translate competencies into clinical practice, aligning assessment strategies, and utilizing systematic curriculum mapping to ensure coherence and comprehensive competency attainment. To synthesize the evidence surrounding competency-based dental education (CBDE) and competency frameworks in postgraduate orthodontic training, a narrative review of the literature was conducted focusing on CBDE, postgraduate orthodontic education, competency domains, curriculum design, assessment strategies, and EPAs. Relevant references were determined from the orthodontic education consensus studies, CBDE literature, and systematic reviews. In total, 10 orthodontic competency areas, 10 POs, and 11 COs were identified methodically. Ten essential EPAs associated with independent orthodontic practice were then created to operationalize these competencies in clinical practice settings. With this in mind, a three-year curriculum was designed, rooted in elements that aligned with CBDE principles to include competency mapping, assessment techniques, and introducing digital technologies to support teaching, learning, and assessment. This proposed framework provides a clear, distinct, and structured solution to training orthodontic specialties, which would enable it to be embraced as a reference structure for the reform in curriculum on an international level as well. Recent evidence supports the call for standardized orthodontic competencies and a competency-based method of assessment to promote uniformity and quality of postgraduate orthodontic education.

## Introduction and background

A structured postgraduate program gives a deeper insight into the operational components of the program. It perhaps provides a template to the course that the educators can use to teach the subject in an effective manner and for students to comprehend every subaspect of it.

The global transition to a competency-based dental education (CBDE) is indicative of a shift away from the traditional model of time-based curriculum and towards an outcome-centered, mastery-oriented training approaches [1,2]. CBDE refers to an educational framework that emphasizes the attainment of clearly defined competencies - knowledge, skills, attitudes, and professional behaviors - required for independent clinical practice. It focuses on learner progression based on demonstrated ability rather than time spent in training. In orthodontics, this transition is especially significant given that this is a fraternity involving a diverse spectrum of subjects from adolescents to adults requiring efficient diagnosis and meticulous execution of planned strategies [3,4]. Perhaps, the specialty’s commitment to measurable clinical proficiency in challenging fields such as diagnosis, biomechanics, and interdisciplinary patient care makes this necessary [5,6].

Within CBDE frameworks, program outcomes (POs) denote the broad competencies that graduates are expected to achieve by the end of the training program, while course outcomes (COs) specify the measurable abilities that students should acquire at the completion of individual courses. Entrustable professional activities (EPAs) are units of professional practice that can be entrusted to a learner once sufficient competence has been demonstrated, thereby linking theoretical competencies to real-world clinical responsibilities.

Standardization of a postgraduate curriculum will help ensure uniformity across institutions. Notwithstanding this changing educational paradigm, substantial diversity still exists in the provision and evaluation of clinical training across structured postgraduate orthodontic programs, necessitating towards the importance of more uniformity in educational outcomes [7-9]. Concurrently, consensus-based studies have begun to delineate the core competencies and the corresponding cognitive levels that are necessary for effective orthodontic education, thereby making a step towards the direction of competency-based curricular reform [5,6].

A recent modified Delphi study identified a comprehensive set of competencies tailored to postgraduate orthodontic training, offering substantial empirical justification for the development and application of structured CBDE frameworks within the specialty [6]. A Delphi study is a systematic, iterative method of achieving consensus among a panel of experts through multiple rounds of questionnaires, with controlled feedback provided between rounds to refine opinions and arrive at agreement on specific topics. This kind of competency-based framework can help students reach the set learning goals in a timely manner [2].

Interdisciplinary orthodontics is a wide field that has not been explored much in terms of its POs and COs. It is therefore essential to take cognizance of the insights of professionals from other specialties too when developing the competency-based curriculum [4].

## Review

Methods

A narrative review was conducted to identify competency domains relevant to orthodontic specialty training. Sources included Delphi-based competency studies [6], reviews of orthodontic postgraduate knowledge delivery [7], foundational work on orthodontic competency [3], and literature on CBDE frameworks in health professions education [1,2]. Themes were synthesized to develop a comprehensive CBDE model. Primary sources included Delphi-based and consensus studies that established competency domains for postgraduate orthodontics education. These studies have been viewed as crucial because of their academic rigor and subject expertise endorsed validation, with these being the linchpin for defining orthodontic competency domains and expected graduate capabilities [6]. The secondary sources comprised narrative reviews that investigated the content, delivery, and evaluation of postgraduate orthodontic education across universities. Reviews like these explored the variations in training models, lacunae in assessment practices, and the need for outcome standardization across the orthodontic curricula [7]. In order to provide a context on the evolution of competency expectations in orthodontics and thus in line with the historical consensus in educational education, foundational literature on orthodontic competency and specialty training standards was included [3]. In addition, research on CBDE and frameworks for outcome-based education from dentistry, along with other allied health professions, was included as a framework for the structural design of the proposed CBDE model. These sources offered a theoretical basis for the incorporation of POs, COs, EPAs, and aligned assessment paradigms [1,2].

The review included literature published between January 2000 and December 2024 to capture both foundational and contemporary developments in competency-based education. Searches were conducted between January 2025 and March 2025. Only articles published in English were considered.

Inclusion criteria comprised the following: (1) studies focusing on postgraduate orthodontic education or competency frameworks, (2) Delphi or consensus-based studies defining competencies, (3) narrative or systematic reviews addressing curriculum design, delivery, or assessment in orthodontics, and (4) literature describing CBDE or outcome-based education frameworks applicable to dental or allied health education. Exclusion criteria included (1) undergraduate-only studies without relevance to postgraduate training, (2) opinion pieces without methodological grounding, (3) studies not available in full text, and (4) articles not directly addressing competency development or curriculum structure.

A total of 50 articles were initially identified through database searching. After removal of duplicates and screening of titles and abstracts, 27 articles were retained for full-text review. Of these, 17 studies met the inclusion criteria and were included in the final synthesis.

Data extraction involved identifying key competency domains, educational frameworks, assessment strategies, and curriculum structures from the selected studies. A thematic mapping process was then undertaken, wherein competencies identified across studies were coded, grouped into domains, and aligned with CBDE principles. These domains were subsequently mapped to POs, COs, and EPAs to construct a structured and coherent CBDE model.

Search Strategy

An extensive search strategy was designed to accumulate literature relevant to CBDE and postgraduate orthodontic training. Multiple electronic databases were searched systematically using predefined keywords and Boolean combinations to collect evidence related to orthodontic competency frameworks, curriculum design, EPAs, and assessment methodologies, ensuring a sturdy foundation for the development of the proposed CBDE model. Table 1 describes a comprehensive review of the search strategies employed for data extraction and in detail characteristics of the studies involved in the proposed framework.

**Table 1:** Search strategy in different databases

Database	Keywords Used
PubMed (MEDLINE)	("Competency-Based Education" OR "Competency-Based Dental Education" OR "Outcome-Based Education" OR "Competency framework") AND ("Orthodontics" OR "Orthodontic education" OR "Postgraduate orthodontic training" OR "Orthodontic residency") AND ("Education, Dental, Graduate" OR "postgraduate dental education") AND ("Entrustable Professional Activities" OR "clinical competence" OR "workplace-based assessment" OR "Mini-CEX" OR "OSCE") (("Competency-Based Education"[MeSH]) OR ("Orthodontics"[MeSH]) OR ("Education, Dental, Graduate"[MeSH]))
Scopus	("competency based education" OR "competency-based dental education" OR "outcome based education" OR "clinical competence") AND ("orthodontic education” OR "postgraduate orthodontic training” OR "orthodontic residency") AND ("dental education" OR "postgraduate dental education" OR "graduate dental education") AND ("entrustable professional activities" OR "workplace based assessment” OR "objective structured clinical examination" OR "mini-CEX" OR “DOPS”))
Embase	('competency based education'/exp OR 'professional competence'/exp OR 'clinical competence'/exp OR 'outcome based education':ti,ab) AND ('dental education'/exp OR 'postgraduate education'/exp) AND ('orthodontics'/exp OR 'orthodontic education':ti,ab) AND ('educational assessment'/exp OR 'objective structured clinical examination'/exp OR 'entrustable professional activities':ti,ab OR 'workplace based assessment':ti,ab)
Cochrane	("competency based education" OR "competency-based dental education" OR "outcome based education") AND ("orthodontic education" OR "postgraduate orthodontic training") AND ("entrustable professional activities" OR "objective structured clinical examination" OR "Mini-CEX")

The databases searched included PubMed/MEDLINE, Scopus, Web of Science, and Google Scholar. Keywords used in various combinations included “competency-based dental education”, “orthodontic education”, “postgraduate orthodontics”, “Delphi study”, “competency framework”, “entrustable professional activities”, “curriculum design”, and “assessment methods”. Boolean operators (AND, OR) were applied to refine the search.

Screening was conducted in two stages: initial title and abstract screening followed by full-text review. 

Competency Domains for Postgraduate Orthodontics

Consistent with the findings of Mahdavifard et al. [6], who mapped out 73 essential competencies organized across 10 domains, 10 core competency domains were identified for postgraduate orthodontic training: evaluation of clinical records and orthodontic diagnosis; evaluation and assessment of growth and development and its impact on treatment planning; biomechanics and orthodontic force systems; preventive and interceptive orthodontics; fixed appliance mechanotherapy; functional appliance therapy and extra oral orthopedics; orthodontic management of orthognathic cases with skeletal discrepancy; clear aligner therapy and the integration of digital technologies, use of advanced treatment modalities such as use of self-ligating brackets and lingual orthodontics; research literacy and evidence-based orthodontic practice; and professionalism, ethics, and effective communication.

Program Outcomes

The proposed POs listed in Table 2 align closely with the core competencies identified in the 2024 modified Delphi study by Mahdavifard et al. and reflect the higher-order cognitive expectations [6].

**Table 2:** POs for postgraduate orthodontic training ABO, American Board of Orthodontics; PO, program outcome

PO Code	PO Description
PO1	Orthodontic diagnosis and treatment planning
PO2	Soft tissue paradigm and soft tissue considerations in treatment plan
PO3	Execution of fixed and removable orthodontic procedures
PO4	Implementation of biomechanics and modifying effectively in orthodontic treatment planning
PO5	Interdisciplinary approach for management of complex, mutilated, or orthognathic cases
PO6	Surgical considerations in management of orthognathic cases
PO7	3D imaging and scanning principles for virtual surgical planning and clear aligner systems, self-ligating brackets, or lingual orthodontics appropriately
PO8	Evaluation and critical appraisal of conducted research
PO9	Demonstrate professionalism and effective communication skills
PO10	Clinical documentation as per ABO standards

Course Outcomes

The COs drafted in Table 3 were formulated to align with the cognitive levels outlined in the orthodontic curriculum consensus study by Ono et al. [4] and the specialty-specific competencies identified by Mahdavifard et al. [6]. These outcomes reflect a progressive development of knowledge, skills, and professional competencies expected of postgraduate orthodontic trainees.

**Table 3:** COs for postgraduate orthodontic training CO, course outcome

CO Code	CO Description
CO1	Collaboration of principles of craniofacial growth and development pertaining to orthodontic diagnosis and treatment planning
CO2	Including clinical photographs, radiographs, and digital imaging for patients with functional shift and advanced orthodontic diagnostic aids for management of patients with airway disorders
CO3	Apply biomechanical principles to orthodontic case management
CO4	Perform clinical procedures including bonding, banding, archwire sequencing, archwire coordination
CO5	Effective handling of preventive and interceptive orthodontic procedures to prevent developing malocclusions
CO6	Functional analysis and use of appropriate functional appliances proficiently in appropriate clinical situations
CO7	Plan and manage clear aligner therapy using conventional and digital workflows including appropriate designing of attachments and power ridges
CO8	Coordinate and manage orthodontic treatment in orthognathic surgery cases
CO9	Evidence-based literature review pertaining to orthodontic practice
CO10	Design, conduct, and complete a postgraduate research thesis
CO11	Communicate effectively with patients, peers, and interdisciplinary team members

Entrustable Professional Activities

EPAs were developed to translate defined orthodontic competencies into observable and assessable clinical tasks, consistent with established competency-based practices in medical and dental education. These EPAs, as listed in Table 4, align with the competency criteria described by Mahdavifard et al. [6] and represent core professional activities expected of postgraduate orthodontic trainees upon completion of training.

**Table 4:** EPAs for postgraduate orthodontic training EPA, entrustable professional activity

EPA Identifier	EPA Description
EPA 1	Conduct a comprehensive orthodontic diagnosis, including clinical and radiographic evaluation
EPA 2	Develop comprehensive and individualized orthodontic treatment plans
EPA 3	Accurate bonding of fixed orthodontic appliances taking cognizance of smile arc protection and soft tissue considerations.
EPA 4	Manage alignment, leveling, and space closure during active orthodontic treatment
EPA 5	Effective management of common orthodontic emergencies
EPA 6	Collaborate effectively in interdisciplinary cases
EPA 7	Plan orthodontic management for orthognathic surgery cases for splint preparation, mock surgery and Virtual Surgical Planning.
EPA 8	Plan, implement, and supervise clear aligner therapy
EPA 9	Conduct, interpret, and disseminate orthodontic research
EPA 10	Design and implement appropriate orthodontic retention protocols

Proposed CBDE Curriculum Structure

**Figure 1:**
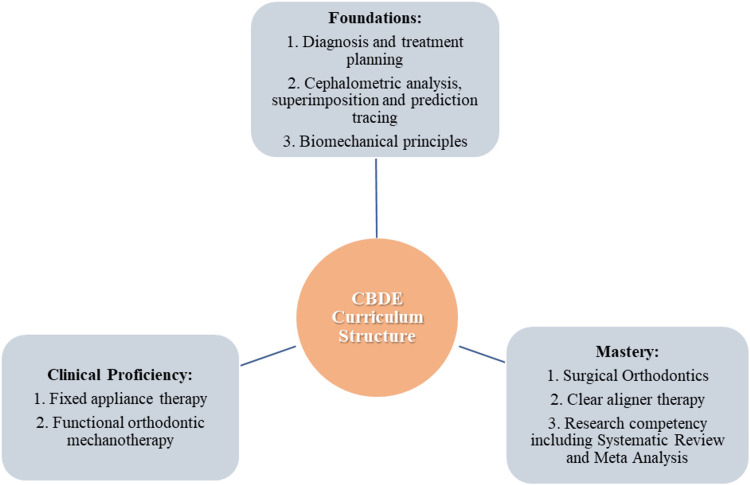
CBDE curriculum structure The figure was created using the SmartArt graphics feature in Microsoft Word. No generative Artificial Intelligence tools were used in the creation of this figure. CBDE, competency-based dental education

Figure 1 depicts the CBDE curriculum structure that is proposed for postgraduate orthodontic training, illustrating a progressive learning model that transitions from foundational basics to clinical proficiency and advanced mastery, ensuring the systematic development of competencies required for independent orthodontic practice.

Assessment Blueprint

In consideration with the recommendations for the standardization of assessment practices in postgraduate orthodontic education [2], a combination of formative and summative assessment strategies was consolidated to support competency-based evaluation.

Formative Assessments

Formative assessments include the following: Mini-Clinical Evaluation Exercise (Mini-CEX), Direct Observation of Procedural Skills (DOPS), case-based discussions, and digital simulation reviews. Mini-CEX is a workplace-based assessment that evaluates clinical reasoning, diagnostic skills, professionalism, and communication through direct observation of real patient encounters [10,11]. DOPS is an assessment method focused on evaluating technical competence and procedural accuracy during specific orthodontic clinical procedures under direct supervision [10]. Case-based discussions are a detailed discussion of clinical cases that can be designed for the assessment of clinical decision-making, application of evidence-based knowledge, and reflective understanding of patient management [12]. Digital simulation reviews is an assessment approach utilizing digital models and virtual simulations for the evaluation of treatment plans, biomechanics, and the use of contemporary orthodontic technologies [13,14].

Summative Assessments

Summative assessments include Objective Structured Clinical Examination (OSCE), which is a structured, station-based examination designed to assess clinical skills, problem-solving ability, and application of theoretical knowledge in standardized clinical scenarios [15], case portfolio (as recommended by consensus-based international orthodontic curricula) [4], evaluation of the postgraduate research thesis, and final clinical examination.

The OSCE is widely regarded as the gold standard for the assessment of clinical competency in health professions education due to its objectivity, reliability, and reproducibility. A well-designed orthodontic OSCE comprises multiple stations, each targeting specific domains of competence. For instance, a bracket positioning station may require candidates to accurately place brackets on a dental model according to prescribed orthodontic principles, thereby assessing precision and understanding of biomechanical concepts. A cephalometric analysis station evaluates diagnostic acumen by requiring the interpretation of radiographic findings and formulation of an appropriate treatment plan. In addition, a wire-bending station assesses technical proficiency and fine motor skills, while a standardized patient interaction station examines communication skills, ethical reasoning, and the ability to obtain informed consent. Each station is typically evaluated using structured checklists and/or global rating scales, which enhance objectivity and minimize examiner bias. Collectively, such well-designed OSCE stations ensure a comprehensive and standardized assessment of clinical competence across cognitive, psychomotor, and affective domains [15].

Table 5 provides an overview of the relationship between POs, COs, and corresponding assessment tools, illustrating how the proposed CBDE framework ensures an all-inclusive and outcome-based evaluation of postgraduate orthodontic competencies.

**Table 5:** Mapping POs, COs, and assessment tools CO, course outcome; DOPS, Direct Observation of Procedural Skills; Mini-CEX, Mini-Clinical Evaluation Exercise; OSCE, Objective Structured Clinical Examination; PO, program outcome

POs	COs	Assessment Tool
PO1	CO1-CO2	Mini-CEX, OSCE
PO3	CO4-CO6	DOPS, case portfolio
PO6	CO8	Orthognathic surgical planning examination
PO7	CO7	Digital aligner planning
PO8	CO9-CO10	Dissertation

Interdisciplinary Orthodontic Outcomes

Interdisciplinary orthodontic outcomes reflect the collaborative role of orthodontics in dental and craniofacial care. As orthodontic treatment involves patients with complex restorative, periodontal, surgical, and developmental needs, effective coordination with allied specialties has become paramount [8]. Defining interdisciplinary outcomes within a competency-based framework ensures that postgraduate orthodontic trainees develop the clinical judgment, communication skills, and collaborative competencies that are required for extensive treatment planning and execution across multidisciplinary clinical settings [8,16].

Assessment of interdisciplinary orthodontic outcomes requires a combination of clinical, functional, esthetic, and patient-reported evaluation tools to capture the full scope of treatment success. Clinically, indices such as the Peer Assessment Rating (PAR) Index and the Index of Orthodontic Treatment Need (IOTN) are widely used to quantify occlusal improvement and treatment necessity, while the American Board of Orthodontics Objective Grading System (ABO-OGS) provides a detailed post-treatment evaluation of alignment, marginal ridges, and occlusal relationships. In interdisciplinary cases - often involving periodontics, prosthodontics, or oral surgery - periodontal parameters (such as probing depth and attachment levels), radiographic analysis (including cone-beam computed tomography for bone and root positioning), and prosthetic outcomes (crown positioning, implant alignment) are also critical. Esthetic assessment tools, including smile analysis and facial harmony indices, help evaluate soft tissue and facial outcomes, while digital tools such as 3D model superimposition enable precise comparison of pre- and post-treatment changes. Additionally, patient-reported outcome measures (PROMs), such as oral health-related quality-of-life questionnaires, are essential for understanding patient satisfaction and functional improvements. Together, these tools provide a comprehensive, evidence-based framework for assessing the success of interdisciplinary orthodontic treatment [8,16].

**Figure 2:**
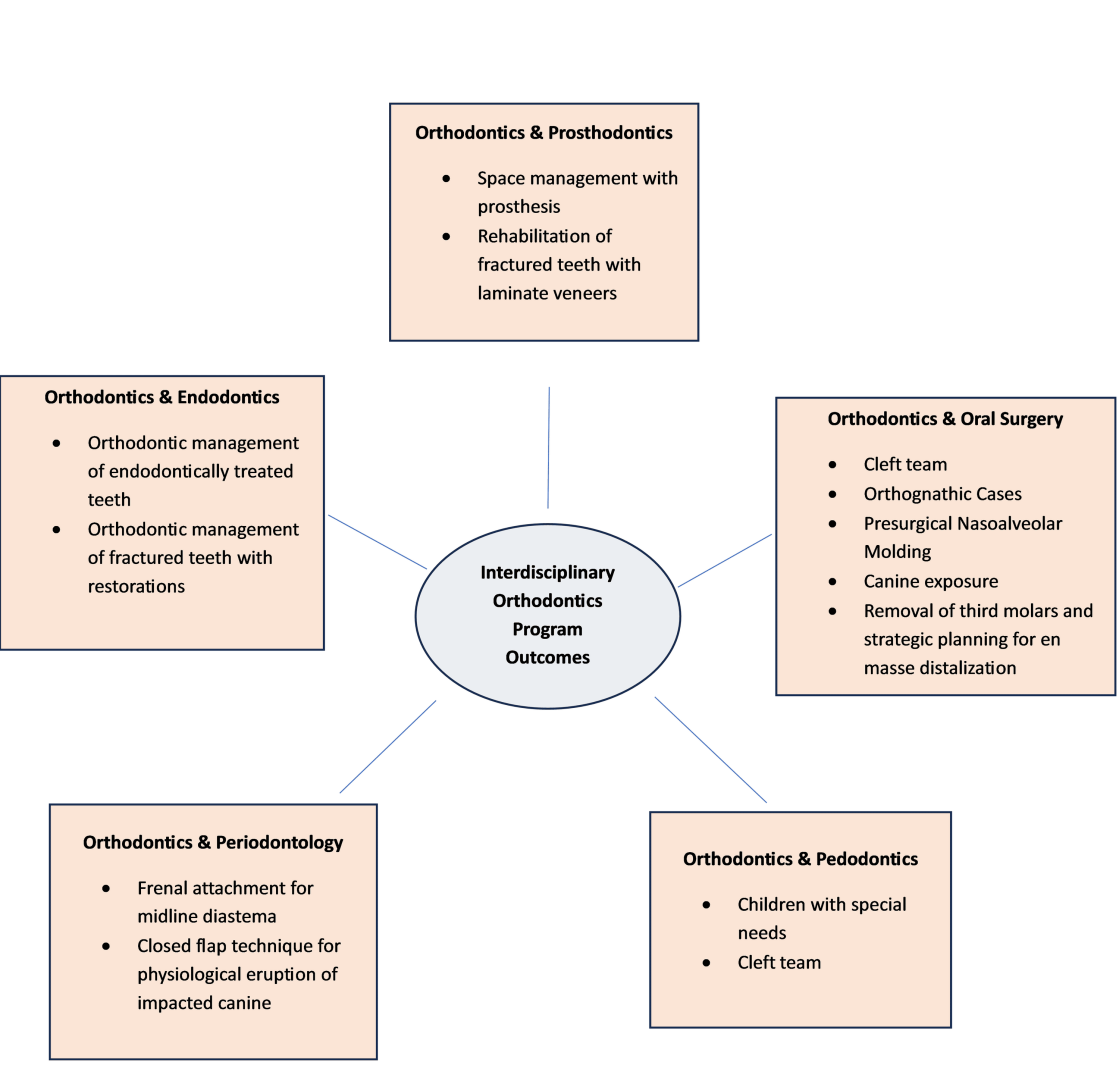
Interdisciplinary orthodontic outcomes within the CBDE framework The figure was created using the SmartArt graphics feature in Microsoft Word. No generative AI tools were used in the creation of this figure. CBDE, competency-based dental education

Figure 2 illustrates key interdisciplinary collaborations between orthodontics and allied dental specialties, highlighting shared clinical responsibilities and coordinated treatment outcomes.

CBDE in postgraduate orthodontic training ensures structured alignment between POs and COs. This mapping links learning activities with clearly defined competencies, enabling measurable, outcome-based education and strengthening clinical, academic, and professional development in orthodontic practice. Table 6 illustrates the mapping of POs, COs, and competency-based outcomes in postgraduate orthodontic training.

**Table 6:** Mapping of POs, COs, and competency-based outcomes in postgraduate orthodontic training ABO-OGS, American Board of Orthodontics Objective Grading System; CBCT, cone-beam computed tomography; CO, course outcome; IOTN, Index of Orthodontic Treatment Need; PAR, Peer Assessment Rating; PO, program outcome

POs	COs	Outcomes (Competency Achievements)
PO1: Apply advanced knowledge of craniofacial growth, development, and orthodontic biomechanics	CO1: Explain principles of craniofacial biology and growth modification	Demonstrates accurate diagnosis and treatment planning based on growth patterns and biomechanical principles
PO2: Diagnose and manage complex orthodontic malocclusions	CO2: Perform comprehensive orthodontic diagnosis using clinical and radiographic tools	Develops precise treatment plans for simple to complex malocclusions with minimal supervision
PO3: Integrate interdisciplinary dental care in treatment planning	CO3: Coordinate orthodontic care with periodontics, prosthodontics, oral surgery, and restorative dentistry	Delivers coordinated treatment plans ensuring optimal functional and esthetic outcomes in multidisciplinary cases
PO4: Perform evidence-based orthodontic treatment procedures	CO4: Apply contemporary orthodontic appliances and techniques	Executes efficient, safe, and evidence-based orthodontic interventions with predictable outcomes
PO5: Demonstrate ethical practice and professionalism in patient care	CO5: Apply ethical principles and professional communication in clinical settings	Maintains patient-centered care, informed consent, and ethical decision-making in all treatments
PO6: Utilize research and critical appraisal in clinical decision-making	CO6: Interpret and apply scientific literature in orthodontic practice	Integrates research evidence into treatment planning and contributes to academic inquiry
PO7: Evaluate treatment outcomes using standardized indices and tools	CO7: Apply outcome assessment tools (e.g., PAR Index, ABO-OGS, IOTN)	Objectively assesses treatment success and continuously improves clinical performance
PO8: Develop competency in digital and advanced orthodontic technologies	CO8: Use digital orthodontic tools including CBCT, digital setups, and aligner systems	Demonstrates proficiency in digital workflows for diagnosis, planning, and appliance design
PO9: Engage in lifelong learning and academic development	CO9: Participate in seminars, case presentations, and continuing dental education	Shows continuous professional development and adaptability to evolving orthodontic practices

**Figure 3:**
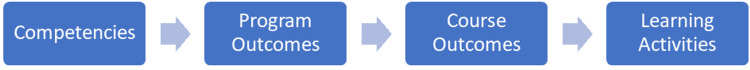
CBDE model for orthodontics The figure was created using the SmartArt graphics feature in Microsoft Word. No generative AI tools were used in the creation of this figure. CBDE, competency-based dental education

Figure 3 illustrates the integrated CBDE model for postgraduate orthodontic training, demonstrating the interrelationship between competency domains, POs, COs, EPAs, and assessment strategies within a structured curriculum framework (aligned with frameworks described by Mahdavifard et al. [6]).

Advances in Teaching-Learning Methods in Orthodontic Postgraduate Education

**Figure 4:**
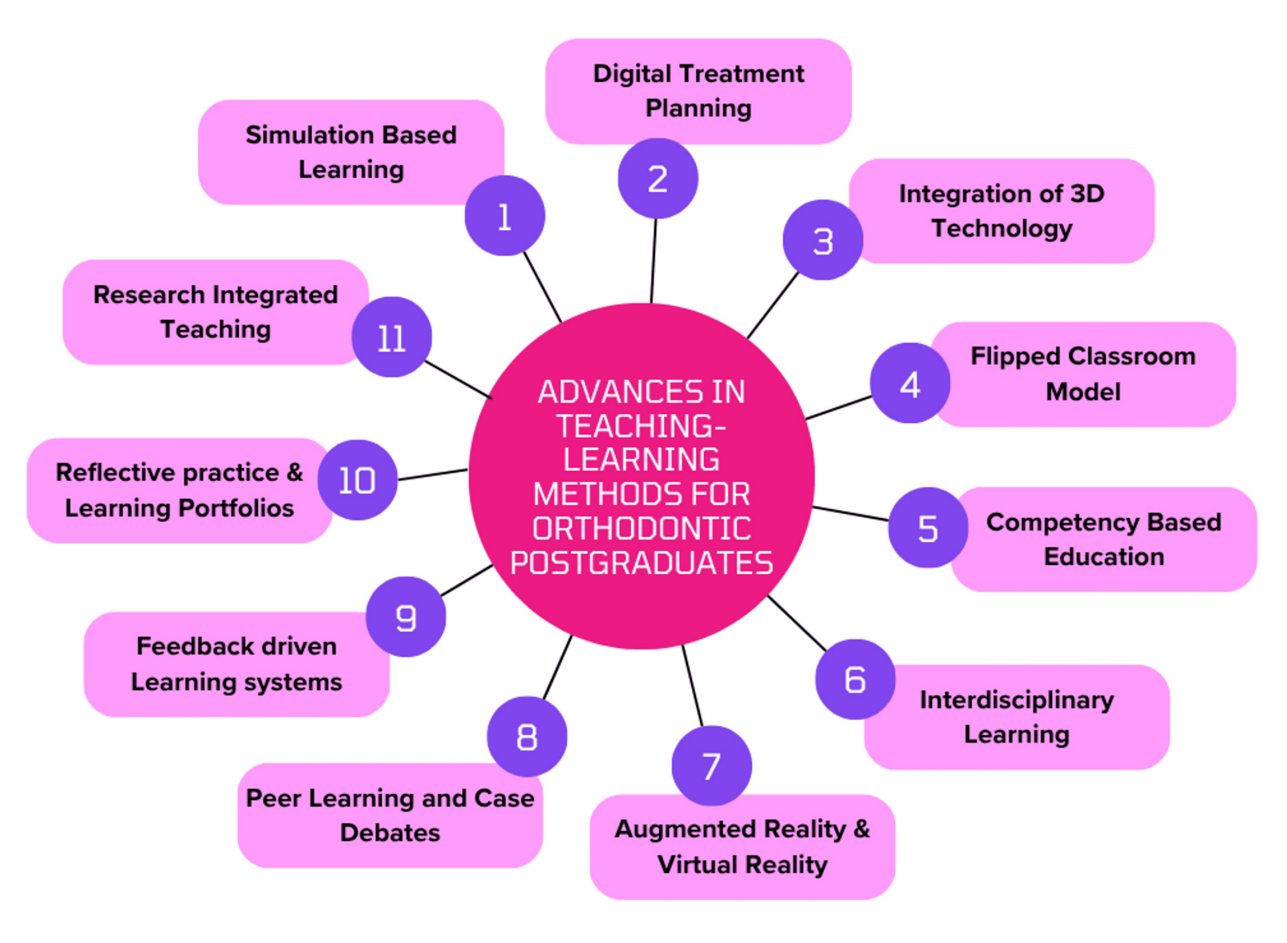
Advances in teaching-learning methods for orthodontic postgraduates The figure was created using Canva. No generative artificial intelligence tools or AI-assisted design features were used in the creation of this figure.

Recent advances in orthodontics postgraduate education emphasize a shift from traditional lecture-based teaching to more interactive, technology-driven, and competency-based approaches. These methods integrate digital tools, simulation, and evidence-based learning to enhance clinical skills, critical thinking, and individualized training outcomes. As illustrated in Figure 4, these modern strategies collectively improve the learning environment by promoting active participation, better diagnostic ability, and more efficient skill acquisition among orthodontic postgraduates.

Digital Treatment Planning

Digital treatment planning can be done using software such as NemoCeph and Dolphin Imaging, which have greatly revolutionized the diagnostic accuracy and treatment outcome prediction in orthodontics. These tools help with detailed cephalometric analysis, digital tracing, and growth prediction, which allow the postgraduates to evaluate skeletal and dental relationships with more precision. In cases where surgical intervention is required, especially orthognathic surgery, these platforms offer virtual surgical planning and simulation of post-treatment outcomes. The postgraduate students develop critical analytical skills and an evidence-based approach to decision-making by comparing various treatment scenarios. This digital integration improves the understanding of complex craniofacial discrepancies and help in easier communication with the patients and interdisciplinary teams [9,13].

Integration of 3D Technology

The integration of three-dimensional (3D) technology, particularly through intraoral scanning and aligner workflows, represents a major advancement in orthodontic education. Intraoral scanners generate highly accurate digital impressions, thus overcoming the limitations of conventional impression techniques. These digital models allow manipulation as per the requirement and can be used for diagnosis, treatment planning, and simulation of tooth movement in all three planes of space. In aligner therapy, stepwise digital setups allow postgraduate trainees to visualize sequential tooth movements and help understand the biomechanics. Incorporation of such digital workflows gears them for modern clinical practice, where aligner-based treatments and CAD/CAM technologies are increasingly prevalent [13,14,17].

Flipped Classroom Model

The flipped classroom model represents a shift from traditional instructive teaching to a more learner-based approach. This method requires students to engage with the lecture material, videos, and readings prior to class, thereby reserving in-class time for interactive sessions such as case discussions, problem-solving exercises, and peer learning. This encourages active participation, deeper understanding, and critical thinking, as the students learn to apply theoretical knowledge to clinical scenarios under the guidance of faculty. The flipped classroom model improves the long-term retention of knowledge and prepares the trainees for independent clinical decision-making by promoting self-directed learning and accountability [14].

Interdisciplinary Learning

Interdisciplinary learning is an important aspect of orthodontic postgraduate training, as comprehensive patient care often requires collaboration with different dental specialties.

In periodontology, procedures such as crown lengthening play a crucial role in improving smile aesthetics and for facilitating a proper placement of brackets by correcting gingival discrepancies. Exposure to these procedures allows orthodontic residents to appreciate the periodontal considerations that influence treatment planning and outcomes [8].

In oral surgery, the management of impacted canines requires close coordination between the orthodontist and the surgeon. Precise timing and biomechanical planning are of utmost importance during the surgical exposure of the impacted tooth, which is followed by orthodontic traction. Similarly, orthognathic surgery is integral to the correction of severe skeletal discrepancies. Postgraduates must understand all the phases of treatment, including pre-surgical orthodontics, surgical planning, and post-surgical finishing, to achieve optimal functional and aesthetic results. Such interdisciplinary exposure enhances their ability to manage complex dentofacial deformities effectively [8].

The principles of occlusion and joint stabilization are introduced to the trainees during the fabrication of splints for temporomandibular joint disorders in prosthodontics. Understanding the design and application of these appliances helps incorporate orthodontic treatment in the management of temporomandibular conditions. Additionally, cases requiring prosthetic rehabilitation often require orthodontic intervention for the creation of space and alignment before the placement of prosthesis. This sequence of treatment highlights the importance of coordinated planning between orthodontists and prosthodontists, particularly in adult patients with missing teeth or compromised dentition [16].

Competency-Based Learning

Competency-based education shifts the focus of orthodontic postgraduate training from simply completing a fixed duration of study to actually mastering essential clinical skills. Students can progress through the program only after demonstrating mastery of clinical competencies such as diagnosis, treatment planning, biomechanics, and case finishing. The objective is to have consistent levels of skill from all graduates, regardless of how quickly they complete each semester or quarter of the program [1-3].

Augmented Reality and Virtual Reality

Augmented reality (AR) and virtual reality (VR) technologies enhance spatial understanding and visualization of orthodontic treatment. Using VR headsets, students can explore 3D craniofacial structures and simulate tooth movement in different planes. For example, a resident can visualize how maxillary expansion affects surrounding structures or how impacted canines are guided into position. AR can overlay digital treatment outcomes onto a patient’s actual face, helping students predict aesthetic results. This immersive learning improves comprehension of complex anatomical relationships [13,17].

Reflective Practice and Learning Portfolios

Encouraging students to maintain reflective portfolios helps them develop critical thinking and self-assessment skills. Each case can be documented stepwise including diagnosis, treatment plan, progress, complications, and outcomes, along with personal observations. For instance, if anchorage loss occurred during treatment, the student can analyze why it happened and how it could have been prevented. Over time, this builds a habit of continuous learning and improvement, which is essential for clinical excellence [12,14].

Research-Integrated Teaching

Integrating research with clinical teaching ensures that students practice evidence-based orthodontics. Instead of treating research as a separate academic requirement, it can be linked to daily clinical work. For example, when the students are planning the treatment of a particular case, they can review current literature on different treatment modalities and apply the most evidence-supported approach. They can also collect and analyze data from their own cases to publish case reports, hence contributing to scientific knowledge while improving their clinical decision-making [9,12].

Peer Learning and Case Debates

Peer learning encourages collaboration and exposure to diverse perspectives. Structured case debates can be particularly effective in orthodontics. For instance, one group of postgraduates may argue in favor of extraction in a borderline crowding case, while another group argues for non-extraction treatment. Faculty can moderate the discussion, highlighting the pros and cons of each approach. This method sharpens analytical skills and helps students appreciate different treatment philosophies [11,12].

Feedback-Driven Learning Systems

Frequent and structured feedback is crucial for skill development in orthodontics. Tools such as Mini-CEX allow faculty to observe students during procedures and provide immediate feedback. For example, after observing a student placing brackets, the instructor can point out errors in positioning and suggest corrections on the spot. This real-time guidance accelerates learning and prevents the reinforcement of incorrect techniques [10,15].

Discussion

The framework provided in this review offers a structured overview of CBDE for postgraduate orthodontic education [2]. In this context, the framework involves competency domains, POs, COs, and EPAs [6,17]. This shift to outcome-oriented orthodontic education suggests solutions for the persistent criticisms of inconsistencies in teaching and evaluation of curriculum in postgraduate orthodontic education [7]. It further reflects the global shift away from orthodontic training on time-based models [1,3].

To facilitate a structured and progressive development of competencies, COs in postgraduate orthodontic training are organized in a year-wise manner. This stratification reflects the principles outlined by the World Federation of Orthodontists (WFO) guidelines [4], which emphasize staged acquisition of knowledge, clinical skills, and professional competence. The progression from foundational understanding in the first year to advanced clinical proficiency and independent practice in the final year ensures effective curriculum mapping, competency assessment, and alignment with outcome-based education frameworks. Table 7 illustrates the year-wise distribution of COs in postgraduate orthodontic training.

**Table 7:** Year-wise distribution of COs in postgraduate orthodontic training CO, course outcome

Year of Training	Domain	COs
Year 1 (foundational stage)	Knowledge and diagnosis	Demonstrate comprehensive understanding of craniofacial growth and development, orthodontic diagnosis, and cephalometric analysis
	Biomechanics	Explain fundamental principles of orthodontic biomechanics and force systems
	Preclinical skills	Perform basic orthodontic procedures on simulation models (e.g., bracket positioning, wire bending) with accuracy
	Materials and techniques	Identify and utilize orthodontic instruments and materials appropriately
	Professional development	Exhibit basic communication skills and understanding of ethical principles in patient care
Year 2 (intermediate stage)	Clinical skills	Formulate and execute treatment plans for routine orthodontic cases using fixed and functional appliances
	Applied biomechanics	Apply biomechanical principles in space closure, leveling, and alignment
	Patient management	Demonstrate competence in patient monitoring, adjustments, and management of treatment progress
	Evidence-based practice	Integrate current evidence into clinical decision-making and treatment planning
	Communication	Communicate effectively with patients and caregivers regarding treatment procedures and outcomes
Year 3 (advanced stage)	Advanced clinical competence	Manage complex malocclusions and interdisciplinary orthodontic cases independently
	Advanced techniques	Apply advanced mechanotherapy, including clear aligners, digital orthodontics, and skeletal anchorage systems
	Critical thinking and research	Critically appraise scientific literature and contribute to research through thesis work
	Professional autonomy	Demonstrate independent clinical decision-making, ethical practice, and leadership skills
	Interdisciplinary care	Collaborate effectively with other dental and medical specialties in comprehensive patient management

One of the greatest strengths of this framework is that it has been grounded on consensus-based evidence, which holds immense value [6]. This has been illustrated by the competency domains corresponding to the different aspects of the nature of the topic of orthodontics, which involve far more than just operative skills, such as analysis skills, biomechanical understanding, coordination, research, participation, and personal conduct [4,6]. This was further made interoperable by connecting the domains to the POs and COs (Tables 2, 3), thus improving the alignment in learning, assessment, and instruction, which is a core component of competency-based learning [2].

The incorporation of EPAs is another major innovation [17]. Competency statements describe the broad knowledge, skills, attitudes, and professional behaviors expected of trainees, whereas EPAs translate these competencies into specific day-to-day clinical tasks that can be directly observed, assessed, and entrusted to the learner in clinical practice [17]. In orthodontic clinical practice, which involves long-term patient management and complex clinical decision-making, EPAs serve as a practical framework for the progressive development of clinical competence by facilitating stepwise delegation of responsibilities as trainees gain experience and proficiency [6,17]. Thus, the EPAs suggested in this framework (Table 4) represent vital processes required of an independent orthodontist and, as such, provide a strengthened link between training and actual clinical practice [6].

Another significant contribution of the proposed model is the importance given to mapping and blueprinting of curricula [5]. By systematically aligning POs and COs with both formative and summative assessment tools (Table 5), the framework encourages holistic assessment of learner progress instead of reliance on isolated examinations [7]. Workplace assessment, case portfolio, OSCEs, and research assessment add value in offering components of holistic assessment of clinical skills, professionalism, and critical thinking, which simply cannot be accurately assessed by a single process and by traditional methods alone [10,11,15].

The proposed competency-based framework is aligned with the recommendations of the World Federation of Orthodontists (WFO), which has established comprehensive guidelines for postgraduate orthodontic education to ensure uniformity and high standards in specialist training worldwide [4]. 

Program Goals and Objectives 

The WFO guidelines emphasize the development of competent orthodontic specialists with strong clinical, academic, and research training. Graduates should be capable of diagnosing and managing all forms of malocclusion, formulating and executing comprehensive orthodontic treatment plans, and managing interdisciplinary as well as complex craniofacial cases. In addition, the program should promote ethical and evidence-based clinical practice while fostering the ability to critically evaluate orthodontic research and scientific literature. 

*Program Duration* 

According to the WFO recommendations, postgraduate orthodontic education should consist of a minimum of 24 months of full-time training, with 36 months being the preferred duration to ensure adequate clinical exposure, completion of research activities, and appropriate patient retention and follow-up. 

Admission Requirements 

The guidelines recommend that applicants possess a basic dental qualification obtained through a minimum of four years of dental education and hold valid dental registration. Preference may be given to candidates with prior general dental practice experience. Selection should be based on academic merit, clinical aptitude, and motivation toward specialty training. 

Clinical Training Requirements 

The WFO advocates structured, full-time clinical training under continuous supervision. Residents should receive sufficient clinical exposure through supervised patient management and should gain experience in fixed and removable appliance therapy, growth modification, interceptive and preventive orthodontics, orthognathic surgery cases, and interdisciplinary as well as craniofacial anomaly management. 

Faculty Requirements 

The guidelines emphasize the importance of qualified orthodontic faculty possessing specialist registration, substantial clinical expertise, and academic and research credentials. A favorable faculty-to-resident ratio is recommended to ensure effective supervision and mentorship. Furthermore, the program director should demonstrate strong leadership in clinical practice, academics, and research. 

*Curriculum Components* 

The curriculum should incorporate advanced orthodontic diagnosis and treatment planning, craniofacial growth and development, biomechanics, biomaterials, orthognathic surgery principles, interdisciplinary dentistry, research methodology, and biostatistics. Academic activities such as journal clubs, seminars, literature reviews, and case presentations are also considered essential components of postgraduate orthodontic education. 

*Research Training* 

The WFO guidelines recommend a mandatory research component within the postgraduate curriculum. Residents should be trained in research methodology, hypothesis formulation, data collection and analysis, and scientific writing, culminating in the presentation and publication of research findings. 

Facilities and Resources 

Adequate infrastructure is considered essential for effective orthodontic training. The guidelines recommend the availability of well-equipped clinical facilities, orthodontic laboratories, technical and research support, academic resources, and access to contemporary diagnostic and treatment technologies to facilitate comprehensive postgraduate education.

This framework also encapsulates how modern orthodontic practice is transitioning [8]. Incorporation of digital technologies, clear aligner therapy, virtual surgical planning, and advanced appliance systems is quite inclusive and prepares the trainees to adapt to these rapidly evolving technologies [14]. Moreover, interdisciplinary orthodontic outcomes address a critical but often overlooked area of postgraduate education, namely reinforcing the orthodontist’s role within multidisciplinary healthcare teams [8,16].

Despite the advantages of this framework, it still has some limitations. As it follows a narrative review, its effectiveness in practical terms depends on the preparedness of the institution, training of faculty members, and availability of resources required to implement it [7]. Certain challenges may arise in uniform implementation of this framework due to variations in clinical experiences, training capabilities of faculty members, and the necessary infrastructure across different institutions [8]. Furthermore, while the framework emphasizes standardization of results, it needs to be sufficiently flexible in terms of regional regulations, independence of the institution, and contextual needs [3].

Future research should focus on piloting and validating the CBDE framework across the diverse postgraduate orthodontic programs [6]. Longitudinal studies on the learning pace of participants, reliability of assessment, faculty perceptions, and patient-related outcomes would provide a substantial amount of evidence on the feasibility and impact of this framework [7]. Ongoing improvements in EPAs and assessment tools with the help of input from stakeholders are expected to enhance the practicality and acceptance of the framework [17].

## Conclusions

In conclusion, this CBDE framework offers a conceptual, evidence-based model to support postgraduate orthodontic training based on competencies, clinical practice, assessment strategies, and curriculum design. By introducing standardization, it may improve the quality, consistency, and accountability of orthodontic specialty training, both nationally and internationally, without losing adaptability.
